# The Grocery Druggists

**Published:** 1902-12

**Authors:** 


### The Grocery Druggists.

IN ALL the Buffalo newspapers recently there appeared a four-column advertisement of a prominent grocery firm which runs a grab-all series of side issues in both its Main street stores, including a drug department. This is unquestionably the approach to the "community of interest" idea, this running together of turnips and tablets and potatoes and pills, to say nothing of peas and plasters. It makes things easy for the customer, for he has only to walk from one end of the store to the other to get material for a Welsh rarebit and the calomel and salines which are usually necessary afterward. The advertisement announced in big letters a "free distribution of full-sized packages" of a queer named doctor's "genuine dynamised" homeopathic preparations. There is then given a list of the pills which may be selected from by the customer who has cut out a coupon which was printed in the advertisement. And this list is a marvel when one considers that these drugs, by the authority of the firm which fathers the drug store in the patch of cabbages, are advertised as "the only scientific medicines in the world." In the list "infantile bronchitis" is given as pneumonia; "scurfs" as "eczema, raw surfaces;" "pimples on the face, postules;" "tonsillitis" is "quinsy;" "consumption" is "hectic fever, debility and tubercles," each of which have a separate medicine which is guaranteed to cure. "Salt rheum" is diagnosticated as "varicose ulcers;" and "felons" are given the distinguished title of "panaritium." But the acme of what Josh Billings was wont to picturesquely term "damphulishness" is reached in "prostratorrhœa" which is merely designated as "discharges," and in "varicocele," a wasting of the "parts." It would be amusing, this grocery store "community of interests," where one may get soda water, candy, smoked and fresh meats and fish, vegetables, cigars, tripe, pickles and have wasted parts restored, were there not elements of the tragic in the pathos of the thought of how many hypochondriacs there are in this town who are eating those "dynamised" pills because they get them free, and how many babies are being stuffed with them because there happens to be a bottle in the house which bears a label guaranteeing certain cure of "infantile bronchitis."

The pity is that the great state of New York permits gratuitous distribution of any old kind of a fake medicine without regard to the disease or condition of the person who receives it.

